# Sunday Rest

**Published:** 1870-09

**Authors:** 


### HALL'S

### JOURNAL OF HEALTH.

Vol. XVII. — SEPTEMBER, 1870. — No. IX.

### SUNDAY REST.

The longest of all the ten commandments is that which explains and defines the nature and object of the Sabbath-day, and the manner of its employment; the great central idea is, that it should be a day of "rest."

Rest for the body or the physical nature.
Rest for the brain or the mental nature.
Rest for the heart or the moral nature.

The body must be rested by a cessation from work or muscular effort.

The brain must be rested by cessation of ordinary mental application.

The heart, the moral nature, must be rested, by a diversion of its activities to subjects belonging more directly to its higher nature.

If you get tired of carrying a bundle on one arm, shift it to the other, and the resting gives the opportunity of accumulating strength for another effort; thus the whole work is carried on all the time, by the alternating of rest and exertion, and it would be impossible without. One set of muscles or activities is rested, by bringing another set into motion.

So as to the brain ; if one set of organs is kept in action all the time, the man becomes a monomaniac; he becomes crazy as to that set of organs. If a man is in great trouble, his friends seek relief for him by diverting his mind; by presenting other objects to it, by which a different set of organs shall be brought into play.

So with our moral nature, our affections, our religious sentiments; to keep them in healthful activity, the objects on which they act, or upon which they go out, must be changed from time to time, or an unwholesome, a destructive effect results. If a man's affections, his loves go out towards money all the time, and exclusively, he becomes a mean, miserable miser, and incompetent to dwell on any subject outside of money. If he thinks of nothing else but religion, he becomes a religious enthusiast, a hard recluse, and unfit for the performance of life's duties. Therefore, to have our moral nature kept in a healthful condition, fitting the man for the proper performance of all his duties, to the state, to society, to the family, and his Maker, his religious sentiments, his social sentiments, his worldly sentiments, must be brought into alternate exercise. Hence the appointment of times and seasons for the cultivation of religious sentiments, as in the public worship of God on the Sabbath day, and in those private internal contemplations which draw the mind off from the world, and engage it on subjects which are spiritual, eternal, and divine.

And as the Sabbath day with its public religious worship, as embodied in praise and prayer, and the reading and exposition of the Bible, offers the best known method of cultivating the religious sentiments, and the opportunity of testifying thus our fealty to God, and our appreciation of his character, his service, and his worship, whatever hinders this, whatever tends to draw, or tempt the mind from these employments, is impolitic, is unwise, is sinful, and to the extent that it is carried out, is destructive to society and religion.

Hence any employment of the hours of the Sabbath day which is a Divine appointment, which thwarts the Divine purposes, will meet with a curse.

LOVE TO GOD, AND LOVE TO MAN, embodies all true religion, and in connection with the express command in reference to the Sabbath day, "In it thou shalt not do any work," it may be fairly inferred that we are to do nothing on the Sabbath day which is not connected with the public worship of God and the private contemplation of religious, spiritual things, except so far as is necessary to deeds of mercy and help to God's creatures, ourselves included.

Whatever does not promote religious contemplation more than remaining at home and engaging in religious reading, reflection, and devotion, is a violation of the Sabbath day.

"SACRED" CONCERTS,

Exhibitions of works of art, open reading-rooms and libraries, walks into the country, frequenting public parks, social visiting, street-walking for exercise, riding out, are all ingenious devices of the wicked one to entice men from the worship of God, and attention to their soul's best interests, under the guise of a false reasoning. As proof, read French History.

The men of the Revolution, for convenience of counting, and to show themselves wiser than God, and knowing, too, that the abolition of the Sabbath day would be an important entering wedge to the annihilation of the Christian religion, determined that there should be no seventh day rest of Bible appointment; and decreed that every tenth day should be a day of rest; but they soon found that it worked badly, and returned to the old order of things in part. That is, a portion of the Sabbath day was spent in religious worship, the forenoon; while the afternoon and night was employed, and has been ever since, in promenades, going to theatres, visiting museums, frequenting public libraries, picture galleries, and the like; and look at the result! A statement comes across the water within a month, that French physicians assert that the acknowledged physical deterioration of the French, especially in Paris, is owing to the want of rest on Sundays. It is no rest of the body, on Sundays to take walks and drives into the country, or in the streets; it is no rest to the brain, to clerks and students, and business men, to be poring over books in libraries, and reading long-winded novels. It is no rest to the moral sentiments, and to the affections, to let them go out on the same objects as on weekdays. But it is a rest to the body to cease to do work, and to sleep as much as the body will take, on the seventh day; it is a rest to the brain, to call it away from the intense workings which engaged it during the week, and it is a rest to our moral and religious nature and sentiment, to divert them on the Sabbath day, as far as possible, from all worldly objects, and let them go out towards what is spiritual, heavenly, and divine.

Thus it is that common sense, reason, physiological law, all unite with the Divine command in ordaining a day of rest for every creature, expressed in words coming from the Almighty mind in wisdom, mercy, and loving kindness —

"REMEMBER THE SABBATH DAY, TO KEEP IT HOLY."

